# Hypocholesterolemia and Inflammatory Biomarkers Act as Predictors of Severe Vitamin D Deficiency in Patients With Crohn’s Disease: A Clinical Analysis of 862 Patients in China

**DOI:** 10.3389/fnut.2022.806887

**Published:** 2022-04-13

**Authors:** Jie Lu, Fei Yu, Jun Huang, Haitao Yu, Fengying Li, Zhi’an Le, Yulan Cheng, Qi Zhang, Guiling Li, Xinyou Xie, Huifang Tang, Jun Zhang

**Affiliations:** ^1^Clinical Laboratory, Sir Run Run Shaw Hospital, Zhejiang University School of Medicine, Hangzhou, China; ^2^Key Laboratory of Precision Medicine in Diagnosis and Monitoring Research of Zhejiang Province, Hangzhou, China; ^3^The Second Affiliated Hospital of Zhejiang Chinese Medical University, Hangzhou, China; ^4^Department of Pharmacology and Sir Run Run Shaw Hospital, Zhejiang University School of Medicine, Hangzhou, China

**Keywords:** vitamin D, Crohn’s disease, dyslipidemia, inflammation, penetrating disease

## Abstract

**Background:**

In this study, we enrolled 862 patients with Crohn’s disease (CD) in China to investigate the correlation between serum vitamin D (SVD) and serum lipids, inflammatory biomarkers, and important clinical parameters.

**Materials and Methods:**

25(OH)D was measured by LS/MS/MS. Correlation analysis, chi-square tests, and logistic regression analysis were performed to determine the correlations between vitamin D and potential risk factors when vitamin D levels were lower than 10 ng/mL or 20 ng/mL.

**Results:**

The incidence of severe vitamin D deficiency (SVD < 10 ng/mL) in patients with CD was significantly higher than that in healthy controls (28.9 vs. 9.5%). Multinomial logistic regression analysis showed that penetrating disease [odds ratio (OR) = 2.18], low levels of high-density lipoprotein cholesterol (HDL) (*OR* = 1.91), high erythrocyte sedimentation rate (*OR* = 1.73), and platelet count (PLT) (*OR* = 2.71) were regarded as predictors of severe vitamin D deficiency, while only PLT (*OR* = 1.90) and HDL (*OR* = 1.76) were considered as predictors of mild vitamin D deficiency (SVD 10–20 ng/mL).

**Conclusion:**

Our results confirm a higher incidence of severe vitamin D deficiency in patients with CD in China and show that vitamin D deficiency could result from the combined effects of penetrating disease, inflammation, and low levels of HDL.

## Introduction

Crohn’s disease (CD), a main type of inflammatory bowel disease (IBD), is characterized by a chronic relapsing inflammation throughout the gastrointestinal (GI) tract (1). CD seems to be caused by complex interactions among genetic, immunological, and environmental risk factors (2), but the specific pathogenesis of CD remains unclear.

Vitamin D is a fat-soluble vitamin. Recent studies have suggested the role of vitamin D in immunomodulation (3). There is crosstalk between hypovitaminosis D and CD (4, 5), and vitamin D has even been proposed as a treatment for IBD (6). Long-term vitamin D deficiency may promote higher disease activity and poor prognosis of CD (7). Moreover, the risk of vitamin D deficiency in CD patients is elevated, which could be a result of the combined effect of factors either unleashed by the inflammation itself or derived from the disease (8–10). Therefore, assessment of these risk factors may help to predict vitamin D deficiency and prevent its negative consequences.

Some evidence has suggested that malnutrition or metabolic disorders, including hypovitaminosis D and dyslipidemia, can be found in most patients with CD (11, 12). In these patients, dyslipidemia, including hypocholesterolemia and hypertriglyceridemia, has been related to the severity of illness (13, 14). The majority of studies have reported a direct positive association between high-density lipoprotein cholesterol (HDL) levels and vitamin D; weak inverse correlations between serum triglyceride (TG) and total cholesterol (TC) levels; and vitamin D status in healthy children and adolescents (15–17). Vitamin D supplementation appeared to have a beneficial effect on reducing serum total cholesterol, LDL cholesterol, and triglyceride levels (18, 19). All these results suggest a relationship between vitamin D and lipid profiles, while data from patients with CD are scarce, and how lipid profiles affect vitamin D remains unclear.

In addition, inflammatory processes can impact the patients’ nutritional status. A large number of studies of patients in the United States, Italy, Germany, Norway, Iran, and South Korea have suggested that low vitamin D levels are associated with important clinical parameters, such as disease activity, endoscopy, and inflammatory history (20–27), while other studies have demonstrated a lack of statistically significant association (28, 29). These conflicting results may be explained by differences in sample size, participants from different regions, or differences between laboratories.

Vitamin D levels in China are lower than expected (30). However, data representing the prevalence of vitamin D deficiency in Chinese patients with CD are unclear (31–33). Therefore, to characterize the vitamin D status of Chinese patients with CD, in this study, we enrolled 862 patients with CD to investigate the relationship of vitamin D with serum lipids, inflammatory biomarkers, and important clinical parameters. Finally, the negative consequences and risk factors for vitamin D deficiency in the progression of CD were determined.

## Materials and Methods

### Patients

From March 2014 to August 2020, a cross-sectional study was performed on a cohort of 862 patients diagnosed with CD from the Department of Gastroenterology of Sir Run Run Shaw Hospital affiliated to the Zhejiang University School of Medicine. Our registry includes demographic, laboratory, endoscopic, radiological, and clinical data of enrolled patients, and is updated routinely through information technology support. The basic demographic data, including age, gender, and vitamin D supplementation history of each subject were recorded. As part of their usual IBD care, patients underwent routine testing for serum 25(OH)D levels on admission. A total of 576 healthy controls were selected based on age, sex, and registration year to ensure similar exposure to healthcare between patients with CD and controls. Healthy controls underwent a general physical examination within 1 year of their corresponding case diagnosis and had normal findings. Controls were evaluated for participation eligibility using the same enrollment and exclusion criteria as those used for patients with CD.

**FIGURE 1:**
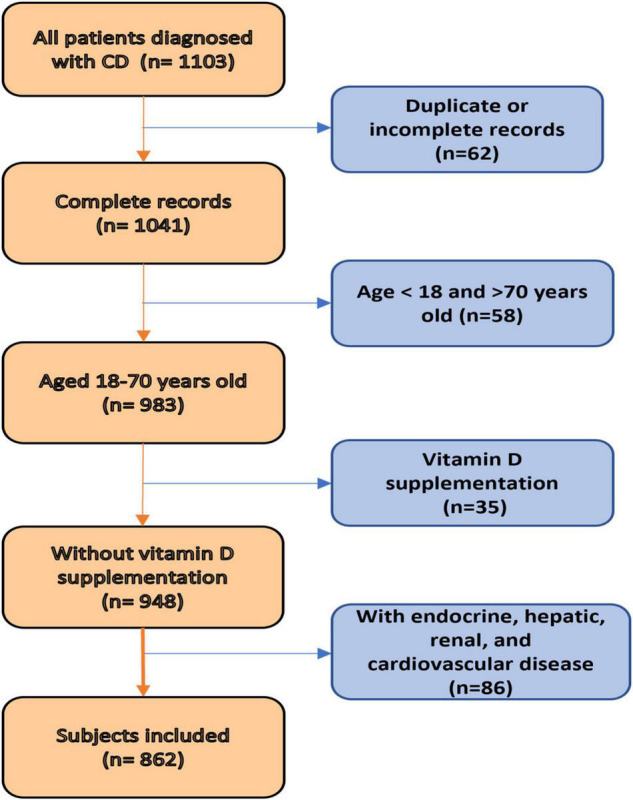
Flowchart presenting the steps of inclusion and exclusion of subjects.

Subjects’ inclusion and exclusion criteria are depicted as a flowchart shown in Figure 1. The inclusion criteria for this study were: age between 18 and 70 years and an established diagnosis of CD based on clinical, endoscopic, and histological criteria. The exclusion criteria included endocrine, hepatic, renal disorders; cardiovascular disease; and the use of vitamin D supplements. After overnight fasting, blood samples were collected from patients with CD and healthy controls for the evaluation of vitamin D, biochemical, and hematological analyses.

### Clinical Status of Patients With Crohn’s Disease

Body mass index (BMI) was calculated at a time when weight had been stable. To evaluate disease activity, we used the Harvey-Bradshaw index (HBI) to divide patients with CD into four groups: remission group (HBI < 5), mild group (HBI 5–7), moderate group (HBI 8–16), and severe group (HBI > 16). Regarding the location of the disease, patients were classified into four groups: L1 (terminal ileal disease ± localized cecal disease), L2 (colonic disease), L3 (ileocolic disease), or L4 (isolated upper disease). The disease behavior of each patient was categorized into three groups: B1 (non-stricturing non-penetrating disease), B2 (stricturing disease), and B3 (penetrating disease). Perianal disease was positive if the patient had any one of the following: skin tag, anal fissure, perianal abscess, or perianal fistula. Surgical history referred to any confirmed, documented surgical procedure necessary for the treatment or management of CD, except surgery related to the existing perianal disease.

### Vitamin D Status Assessment

25(OH)D, including 25(OH)D2 and 25(OH)D3, was measured using AB Sciex Triplie QuadTM4500MDA liquid chromatography tandem mass spectrometry system (LC–MS/MS) (AB Sciex Pte. Ltd., Singapore). Sample preparation includes protein precipitation and liquid–liquid extraction. 25-Hydroxyvitamin D2-IS (25-OH-VD2-IS) and 25 hydroxyvitamin D3-IS (25-OH-VD3-IS) are recommended as internal labels. Standard curves for 25(OH)D2 and 25(OH)D3 were created using separate calibrators (Desaisi, Zhejiang, China) for 25(OH)D2 and 25(OH)D3. During measurement, two levels of quality controls (Desaisi, Zhejiang, China) were used. The standard curves consistently demonstrated excellent correlation coefficient (*r*^2^ > 0.990) and were generated for analysis of 25(OH)D analytes. The analytical linear range and imprecision of the methods were 2.59–109 ng/mL and CV < 15% for 25(OH)D2, 4.72–161 ng/mL and CV < 15% for 25(OH)D3. 25(OH)D is involved in the External Quality Assessment Program of the Chinese National Center for Clinical Laboratories.

According to Clinical Practice Guidelines from the US Endocrine Society, vitamin D deficiency was defined as 25(OH)D levels less than 20 ng/mL. Values below 10 ng/mL indicate severe vitamin D deficiency (34).

### Biochemical and Hematological Analysis

Levels of TGs, TC, HDL, low-density lipoprotein cholesterol (LDL), VLDL, lipoprotein a [Lp(a)], non-esterified fatty acid, high-sensitivity C-reactive protein (hs-CRP), and serum amyloid A (SAA) in the serum samples were quantified using a C-16000 plus biochemistry analyzer (Abbott, Tochigi, Japan). According to a standard protocol, erythrocyte sedimentation rate (ESR) was measured using the MONTIOR 100 auto ESR analyzer (VITAL, Forli, Italy).

The red blood cell (RBC) counts, hemoglobin (Hb), white blood cell (WBC) count, absolute values of different leukocytes (neutrophils, lymphocytes, monocytes, eosinophils, and basophils), and platelet count (PLT) in whole blood samples were quantified using a Beckman Coulter LH 780 hematology analyzers (Beckman Coulter, CA, United States). The platelet-to-lymphocyte ratio (PLR), neutrophil-to-lymphocyte ratio (NLR), monocyte-to-lymphocyte ratio (MLR), and systemic immune inflammation index (SII) were calculated according to neutrophil, lymphocyte, monocyte, and PLTs.

### Statistical Analysis

All analyses were performed using SPSS 20.0 (IBM Corporation, Armonk, NY, United States). The continuous variables are presented as medians and 25th and 75th percentiles. The Kolmogorov-Smirnov test and one-way ANOVA were used to compare quantitative variables among the study groups. Multiple comparisons were performed using the Tamhane’s T2 or Fisher’s least significant difference (LSD) tests. In addition, Spearman’s rho coefficient was used to evaluate the correlations between the measured variables. Categorical parameters are displayed as numbers (%) and were analyzed by the chi-square test. Finally, we used multinomial logistic regression analysis to estimate the odds ratio (OR). Values of *p* < 0.05 were considered statistically significant.

## Results

### Study Cohort Characteristics

A total of 862 patients with CD and 567 healthy controls were recruited for this study. Table 1 illustrates the characteristics of the patients in the two groups. Patients with CD had lower median values of TGs, TC, HDL, LDL, VLDL, RBC, and Hb and higher median values of Lp(a), WBC, PLT, SAA, ESR, and hs-CRP than the healthy controls (*p* < 0.05). BMI, disease activity, and some important clinical information are described in Table 2. Among the patients, 58.1% were men, 41.9% were women, 51.0% had mild to severe disease activity, 49% were in remission, and 42.6% had a stricturing or penetrating disease. A total of 13.4% of patients were overweight. Ileocolic disease was observed in 47.4% of patients and 29.1% of patients had perianal disease.

**TABLE 1:** Characteristics of the CD and healthy control groups.

Parameter	CD (*n* = 862)	HC (*n* = 576)	*p*-value
Age, y, mean ± SD	33 ± 13	34 ± 11	0.526
Male sex, n (%)	593 (68.8%)	336 (58.2%)	–
TG, mmol/L	1.06 (0.81, 1.34)	1.37 (0.96, 2.05)	0.000
TC, mmol/L	3.60 (3.11, 4.23)	4.95 (4.37, 5.61)	0.000
HDL, mmol/L	0.89 (0.76, 1.10)	1.27 (1.08, 1.51)	0.000
LDL, mmol/L	1.97 (2.21, 1.38)	2.84 (2.34, 3.42)	0.000
VLDL, mmol/L	0.39 (0.19, 0.62)	0.58 (0.39,1.04)	0.002
Lpa, mg/dL	27.0 (16.0, 47.3)	8.0 (5.7, 20.0)	0.000
NEFA, μmol/L	324.0 (213.0, 482.0)	370.0 (279.0, 482.0)	0.119
hs-CRP, mg/L	13.5 (3.2, 32.3)	0.8 (0.4, 1.6)	0.000
ESR, mm/hr	16.0 (7.0, 31.0)	7.0 (4.0, 12.0)	0.000
SAA, mg/L	36.40 (6.93, 131.58)	3.10 (1.20, 6.13)	0.000
WBC, × 10^9^/L	6.05 (4.70, 7.70)	5.30 (4.50, 6.30)	0.000
NE, × 10^9^/L	4.00 (2.92, 5.41)	3.05 (2.50, 3.81)	0.000
LY, × 10^9^/L	1.26 (0.95, 1.70)	1.67 (1.37, 2.00)	0.000
MO, × 10^9^/L	0.45 (0.32, 0.60)	0.35 (0.28, 0.45)	0.000
PLT, × 10^9^/L	277.0 (217.0, 345.0)	207.0 (174.0, 242.0)	0.000
RBC, × 10^12^/L	5.82 (3.25, 8.52)	4.71 (4.38, 5.03)	0.000
Hb, g/L	122.0 (108.0, 138.0)	145.0 (134.0, 156.8)	0.000

*TG, triglycerides; TC, total cholesterol; HDL, high-density lipoprotein cholesterol; LDL, low-density lipoprotein cholesterol; hs-CRP, high-sensitive C-reactive protein; WBC, white blood cell; NE, neutrophils; LY, lymphocytes; MO, monocytes; PLT, platelet counts; RBC, red blood cell; Hb, hemoglobin.*

**TABLE 2:** Demographic and phenotypic characteristics of patients with CD.

Parameters	No. (%)
Age (years),mean ± SD	33 ± 13
Male sex (%)	500 (58.1)
**BMI (%)**	
Underweight: <18.5 Kg/m^2^	316 (36.7)
Normal weight: 18.5–23.9 Kg/m^2^	430 (49.9)
Over weight: ≥24.0 Kg/m^2^	115 (13.4)
**HBI (%)**	
Remission: <5	421 (49.0)
Mild: 5–7	276 (32.1)
Moderate to Severe: ≥8	164 (18.9)
**Disease location (%)**	
L1: ileal	269 (31.2)
L2: colonic	93 (10.9)
L3: ileocolonic	408 (47.4)
L4: isolated upper GI	2 (0.2)
L1 + L4; L3 + L4: with upper GI modifier	89 (10.3)
**Disease behavior (%)**	
B1: inflammation	494 (57.4)
B2: stricturing	207 (26.5)
B3: penetrating	89 (13.8)
B2 + B3: stricturing and penetrating	20 (2.3)
**Perianal disease (%)**	250 (29.1)
**Surgery history (%)**	146 (17.0)
**Complication (%)**	309 (35.9)

*BMI, body mass index; HBI, Harvey-Bradshaw index; GI, gastrointestinal.*

### Vitamin D Status in Patients With Crohn’s Disease and Healthy Controls

**FIGURE 2:**
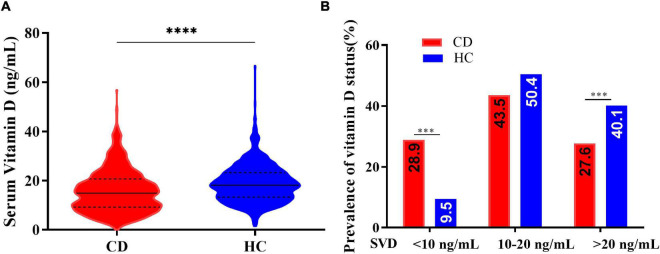
**(A)** Serum vitamin D concentrations in patients with Crohn’s disease (CD) and healthy controls (HC) (Kolmogorov-Smirnov test, ^****^p < 0.0001). **(B)** Vitamin D status in patients with Crohn’s disease (CD) and healthy controls (HC) (chi-square test, ^***^p < 0.005).

The concentrations of serum 25(OH)D were lower in patients with CD than in healthy controls (median: 15.00 vs. 18.15 ng/mL, *p* < 0.001). Vitamin D deficiency (SVD < 20 ng/mL) was present in 72.4% of the patients with CD and severe vitamin D deficiency (SVD < 10 ng/mL) was present in 28.9% of patients. These proportions were markedly higher than those in the healthy control group (59.9 and 9.5%, respectively) (Figure 2).

### Correlation Between Vitamin D Status and Clinical Status/Parameters

**FIGURE 3:**
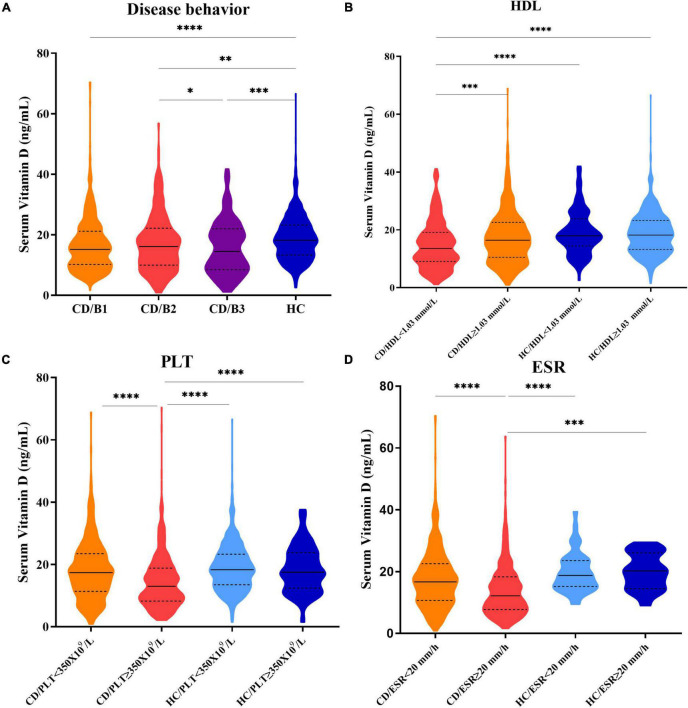
**(A)** Serum vitamin D levels in patients with CD with B1, B2, B3 disease behaviors, and healthy control groups. **(B)** Serum vitamin D levels in CD/HDL < 1.03 mmol/L; CD/HDL ≥ 1.03 mmol/L; HC/HDL < 1.03 mmol/L; HC/HDL ≥ 1.03 mmol/L groups. **(C)** Serum vitamin D levels in CD/PLT < 350 × 10^9^/L; CD/PLT ≥ 350 × 10^9^/L; HC/PLT < 350 × 10^9^/L; HC/PLT ≥ 350 × 10^9^/L groups. **(D)** Serum vitamin D levels in CD/ESR < 20 mm/h; CD/ESR ≥ 20 mm/h; HC/ESR < 20 mm/h; HC/ESR ≥ 20 mm/h groups (one-way ANOVA analysis, *p < 0.05, ^**^p < 0.01, ^***^p < 0.005, ^****^p < 0.0001).

The serum vitamin D (SVD)concentrations were significantly lower in patients with CD with HDL < 1.03 mmol/L, ESR ≥ 20 mm/h, or PLT ≥ 350 × 10^9^/L groups than in patients with HDL ≥ 1.03 mmol/L, ESR < 20 mm/h, or PLT < 350 × 10^9^/L groups (median: 13.53 vs. 16.42 ng/mL; 12.20 vs. 16.71 ng/mL; 13.00 vs. 17.39, respectively, *p* < 0.0001). Patients with CD with B1 (non-stricturing non-penetrating disease), B2 (stricturing disease), or B3 (penetrating disease) had lower levels of SVD than healthy controls. However, no statistically significant differences in vitamin D levels were observed (*p* > 0.05) between healthy controls with different HDL, ESR, and PLT statuses. Interestingly, there were also no statistically significant differences in SVD levels between patients with CD with ESR < 20 mm/h, PLT < 350 × 10^9^/L, or HDL ≥ 1.03 mmol/L groups and healthy controls (Figure 3).

**FIGURE 4:**
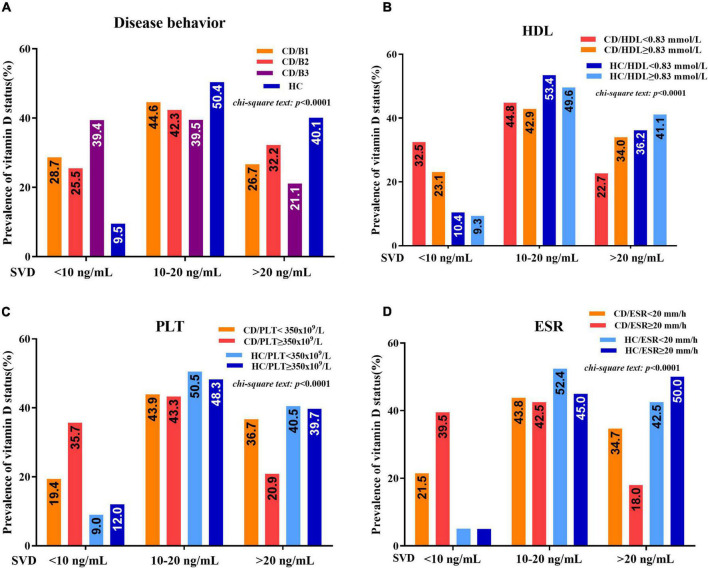
**(A)** Vitamin D status (percentage of serum vitamin D (SVD) <10 ng/mL, 10–20 ng/mL, and >20 ng/mL) between patients with CD and healthy controls concerning different disease behaviors (B1, B2, and B3). **(B)** Vitamin D status between patients with CD and healthy controls concerning HDL < 1.03 mmol/L and HDL ≥ 1.03 mmol/L. **(C)** Vitamin D status between patients with CD and healthy controls concerning PLT < 350 × 10^9^/L and PLT ≥ 350 × 10^9^/L. **(D)** Vitamin D status between patients with CD and healthy controls concerning ESR < 20 mm/h and ESR ≥ 20 mm/h (chi-square test).

Statistically significant associations were observed between vitamin D deficiency and some disease-related parameters, including disease behaviors, HDL, ESR, or PLT in patients with CD, but not in healthy controls. The prevalence rates of severe vitamin D deficiency in CD patients with penetrating disease (B3), HDL < 1.03 mmol/L, ESR ≥ 20 mm/h, or PLT ≥ 350 × 10^9^/L groups were significantly higher than those in patients with HDL ≥ 1.03 mmol/L, ESR < 20 mm/h, or PLT < 350 × 10^9^/L groups (B3:B1: 39.4 vs. 28.7%; HDL: 32.5 vs. 23.1%; ESR: 39.5 vs. 21.5%; PLT: 35.7 vs. 19.4%) (Figure 4).

The differences in lipid and inflammatory parameters among three different vitamin D statuses are shown in Table 3. The levels of TC, HDL, LDL, and VLDL were significantly lower in the SVD < 20 ng/mL group than in the SVD > 20 ng/mL group (*p* < 0.05). The levels of hs-CRP, ESR, SAA, and PLT in the SVD < 10 ng/mL group were significantly higher than those in the SVD 10–20 ng/mL group and the SVD > 20 ng/mL group (*p* < 0.05). Compared to the SVD > 20 ng/mL group, RBCs and Hb decreased significantly only in the SVD < 10 ng/mL group.

**TABLE 3:** Parameter variables among different three vitamin D statuses in patients with Crohn’s disease.

Parameter	SVD < 10 ng/mL (*n* = 249)	SVD 10–20 ng/mL (*n* = 375)	SVD > 20 ng/mL (*n* = 238)	*p*-value
HBI	5.0 (3.0, 7.0)	5.0 (3.0, 6.0)	4.0 (2.0, 6.0)	0.066
BMI, Kg/m^2^	19.38 (17.50, 21.34)	19.33 (17.53, 22.08)	19.77 (17.83, 22.48)	0.254
TG, mmol/L	1.05 (0.81, 1.31)	1.06 (0.80, 1.31)	1.03 (0.82, 1.36)	0.478
TC, mmol/L	**3.48 (2.99, 4.09)^a^**	**3.53 (3.08, 4.10)^b^**	3.86 (3.27, 4.35)	0.003
HDL, mmol/L	**0.86 (0.74, 1.03)^a^**	**0.88 (0.76, 1.08)^b^**	0.97 (0.80, 1.17)	0.000
LDL, mmol/L	**1.89 (1.51, 2.28)^a^**	**1.96 (1.59, 2.35)^b^**	2.06 (1.72, 2.52)	0.005
VLDL, mmol/L	**0.33 (0.16, 0.54)^a^**	**0.39 (0.18, 0.59)^b^**	0.46 (0.25, 0.64)	0.027
Lpa, mg/dL	30.4 (18.8, 52.3)	26.0 (16.0, 46.0)	27.0 (16.0, 50.0)	0.340
NEFA, μmol/L	348.0 (237.0, 516.0)	318.0 (215.5, 461.0)	303.0 (174.8, 454.5)	0.257
hs-CRP, mg/L	**18.80 (6.60, 46.6)^a,c^**	**13.80 (3.75, 25.15)^b^**	7.50 (1.60, 24.40)	0.000
ESR, mm/hr	**23.0 (12.0, 38.5)^a,c^**	**23.50 (9.00, 34.50)^b^**	10.50 (5.00, 22.25)	0.000
SAA, mg/L	**59.90 (15.40, 142.90)^a,c^**	**42.70 (7.00, 131.70)^b^**	14.65 (4.40, 88.08)	0.000
RBC, × 10^12^/L	**4.25 (3.78, 4.67)^a,c^**	4.44 (3.90, 4.91)	4.58 (4.08, 4.92)	0.002
Hb, g/L	**121.0 (107.0, 140.0)^a^**	124.0 (109.0, 141.0)	131.0 (113.0, 144.0)	0.025
WBC, × 10^9^/L	6.20 (4.90, 8.15)	6.00 (4.70, 7.70)	5.70 (4.40, 7.00)	0.578
NE, × 10^9^/L	**4.20 (3.10, 5.81)^a^**	4.00 (2.95, 5.50)	3.72 (2.63, 4.82)	0.001
LY, × 10^9^/L	1.24 (0.93, 1.70)	1.30 (0.97, 1.70)	1.24 (0.96, 1.70)	0.204
MO, × 10^9^/L	**0.47 (0.34, 0.68)^a^**	**0.49 (0.34, 0.60)^b^**	0.39 (0.30, 0.56)	0.019
PLT, × 10^9^/L	**310.0 (242.5, 408.5)^a,c^**	**277.0 (218.0, 339.0)^b^**	241.0 (190.0, 305.0)	0.000
NLR	**3.33 (2.30, 5.15)^a^**	2.96 (2.08, 4.63)	2.92 (2.04, 3.98)	0.049
MLR	**0.39 (0.27, 0.55)^a^**	**0.38 (0.26, 0.50)^b^**	0.30 (0.22, 0.45)	0.041
PLR	**256.06 (178.17, 357.07)^a,c^**	**214.46 (150.75, 317.07)^b^**	190.00 (130.04, 274.75)	0.000
SII	**1043.4 (682.2, 1790.9)^a,c^**	**827.3 (512.3, 1425.0)^b^**	676.6 (433.2, 1189.3)	0.000

*^a^SVD < 10 ng/mL group vs. SVD > 20 ng/mL group, p < 0.05.*

*^b^SVD 10–20 ng/mL group vs. SVD > 20 ng/mL group, p < 0.05.*

*^c^SVD < 10 ng/mL group vs. SVD 10–20 ng/mL group, p < 0.05.*

*p < 0.05 was considered statistically significant (indicated in bold).*

*SVD, serum vitamin D; HBI, Harvey-Bradshaw index; BMI, body mass index; TG, triglycerides; TC, total cholesterol; HDL, high-density lipoprotein cholesterol; LDL, low-density lipoprotein cholesterol; VLDL, very low-density lipoprotein cholesterol; Lpa, lipoprotein a; NEFA, non-esterified fatty acid; hs-CRP, high-sensitive C-reactive protein; ESR, erythrocyte sedimentation rate; SAA, serum amyloid A; WBC, white blood cell; NE, neutrophils; LY, lymphocytes; MO, monocytes; PLT, platelet counts; NLR, the neutrophil-to-lymphocyte ratio; MLR, monocyte-to-lymphocyte ratio; PLR, platelet-to-lymphocyte ratio; SII, systemic immune inflammation index.*

The data in Table 4 suggested that the serum levels of vitamin D were significantly positively correlated with TC (*r* = 0.100, *p* = 0.009), HDL (*r* = 0.143, *p* = 0.000), LDL (*r* = 0.080, *p* = 0.038), and VLDL (*r* = 0.104, *p* = 0.005) in patients with CD. The serum levels of vitamin D were significantly negatively correlated with disease activity [HBI (*r* = −0.110, p = 0.002)] and inflammatory biomarkers, such as hs-CRP (*r* = −0.143, *p* = 0.000), ESR (*r* = −0.200, *p* = 0.000), SAA (*r* = −0.150, *p* = 0.000), PLT (*r* = −0.267, *p* = 0.000), PLR (*r* = −0.180, *p* = 0.000), and SII (*r* = −0.183, *p* = 0.000) in patients with CD. However, there were no statistically significant correlations between vitamin D levels and these parameters (except PLT) in the healthy controls.

**TABLE 4:** Correlation of serum 25(OH)D concentration with lipid profiles and inflammatory biomarkers.

Parameters	CD		HC	
	r	*p*-value	r	*p*-value
Age, years	**0.140**	**0**.**000**	**0.140**	**0**.**001**
HBI	**−0.110**	**0**.**002**	**–**	**–**
BMI, Kg/m^2^	0.042	0.229	0.030	0.329
TG, mmol/L	0.030	0.423	0.066	0.114
TC, mmol/L	**0.100**	**0**.**009**	0.001	0.987
HDL, mmol/L	**0.143**	**0**.**000**	–0.050	0.230
LDL, mmol/L	**0.080**	**0**.**038**	0.010	0.810
VLDL, mmol/L	**0.104**	**0**.**005**	–0.193	0.344
Lpa, mg/dL	–0.055	0.133	–0.145	0.338
NEFA, μmol/L	–0.015	0.699	–0.107	0.529
hs-CRP, mg/L	**−0.143**	**0**.**000**	0.003	0.936
ESR, mm/hr	**−0.200**	**0**.**000**	0.037	0.802
SAA, mg/L	**−0.150**	**0**.**000**	–0.073	0.788
RBC, × 10^12^/L	**0.112**	**0**.**002**	0.049	0.241
Hb, g/L	0.061	0.089	0.072	0.084
WBC, × 10^9^/L	–0.046	0.187	0.009	0.835
NE, × 10^9^/L	**−0.125**	**0**.**000**	0.002	0.963
LY, × 10^9^/L	–0.046	0.184	–0.022	0.593
MO, × 10^9^/L	**−0.100**	**0**.**009**	0.007	0.866
PLT, × 10^9^/L	**−0.267**	**0**.**000**	**−0.083**	**0**.**047**
NLR	**−0.100**	**0**.**008**	–0.018	0.670
MLR	**−0.080**	**0**.**022**	–0.003	0.935
PLR	**−0.180**	**0**.**000**	–0.050	0.234
SII	**−0.183**	**0**.**000**	–0.045	0.278

*P < 0.05 was considered statistically significant (indicated in bold). SVD, serum vitamin D; HBI, Harvey-Bradshaw index; BMI, body mass index; TG, triglycerides; TC, total cholesterol; HDL, high-density lipoprotein cholesterol; LDL, low-density lipoprotein cholesterol; VLDL, very low-density lipoprotein cholesterol; Lpa, lipoprotein a; NEFA, non-esterified fatty acid; hs-CRP, high-sensitive C-reactive protein; ESR, erythrocyte sedimentation rate; SAA, serum amyloid A; RBC, red blood cell; Hb, hemoglobin; WBC, white blood cell; NE, neutrophils; LY, lymphocytes; MO, monocytes; PLT, platelet counts; NLR, the neutrophil-to-lymphocyte ratio; MLR, monocyte-to-lymphocyte ratio; PLR, platelet-to-lymphocyte ratio; SII, systemic immune inflammation index.*

### Logistic Regression Analysis

First, we used univariate logistic regression to generate the full model for comparison. The probability of vitamin D deficiency or severe vitamin D deficiency in each group was separately analyzed by binary univariate logistic regression. Table 5 showed that HBI ≥ 8; ileocolonic (L3); penetrating disease; low levels of TC, HDL-C, and LDL-C; and high levels of ESR, hs-CRP, SAA, WBC, and PLT were considered as independent risk factors for severe vitamin D deficiency in patients with CD. Low levels of HDL and high levels of ESR, hs-CRP, SAA, and PLT were considered independent risk factors for mild vitamin D deficiency.

**TABLE 5:** Logistic regression.

Parameter	SVD < 10 ng/mL vs. SVD > 20 ng/mL				SVD 10–20 ng/mL vs. SVD > 20 ng/mL			
	Full model		Reduced model		Full model		Reduced model	
	OR (95% CI)	*p*-value	OR (95% CI)	*p*-value	OR (95% CI)	*p*-value	OR (95% CI)	*p*-value
**BMI (%)**								
>24	Ref.				Ref.			
≥24	**0**.**56 (0.32, 0.96)**	**0**.**035**			0.71 (0.45, 1.14)	0.157		
**Disease activity**								
HBI < 5	Ref.				Ref.			
HBI 5–7	1.19 (0.77, 1.84)	0.437			1.27 (0.86, 1.88)	0.236		
HBI ≥ 8	**1.89 (1.12, 3.18)**	**0**.**017**			1.49 (0.93, 2.44)	0.119		
**Disease location**								
L1: ileal	Ref.				Ref.			
L2: colonic	1.39 (0.69, 2.80)	0.363			1.27 (0.68, 2.37)	0.462		
L3: ileocolonic	**1.54 (1.01, 2.33)**	**0**.**044**			1.07 (0.73, 1.52)	0.740		
L4: upper GI	1.43 (0.73, 2.80)	0.294			1.47 (0.81, 2.66)	0.200		
**Disease behavior**								
B1: inflammation	Ref.		Ref.		Ref.			
B2: stricturing	0.72 (0.48, 1.11)	0.139	0.57 (0.34, 0.99)	0.051	0.73 (0.50, 1.05)	0.093		
B3: penetrating	**1.89 (1.05, 3.43)**	**0**.**035**	**2.18 (1.08, 4.37)**	**0**.**029**	1.03 (0.56, 1.87)	0.936		
**Perianal disease**								
Without	Ref.				Ref.			
With	1.37 (0.92, 2.06)	0.126			1.19 (0.81, 1.73)	0.375		
**Complication**								
Without	Ref.				Ref.			
With	1.02 (0.70, 1.48)	0.923			1.38 (0.98, 1.94)	0.063		
**TG (mmol/L)**								
<1.70	Ref.				Ref.			
≥1.70	0.83 (0.47, 1.47)	0.529			0.79 (0.48, 1.32)	0.371		
**TC (mmol/L)**								
≥3.0	Ref.				Ref.			
<3.0	**2.02 (1.24, 3.30)**	**0**.**005**			1.42 (0.89, 2.25)	0.147		
**HDL (mmol/L)**								
≥1.03	Ref.		Ref.		Ref.		Ref.	
<1.03	**2.43 (1.60, 3.69)**	**0**.**000**	**1.91 (1.14, 3.19)**	**0**.**014**	**1.78 (1.24, 2.55)**	**0**.**002**	**1.76 (1.14, 2.72)**	**0**.**011**
**LDL (mmol/L)**								
≥1.89	Ref.				Ref.			
<1.89	**1.78 (1.20, 2.63)**	**0**.**004**			1.29 (0.91, 1.85)	0.156		
**hs-CRP (mg/L)**								
<5.0	Ref.				Ref.			
≥5.0	**2.90 (1.93, 4.38)**	**0**.**000**			**1.49 (1.06, 2.10)**	**0**.**022**		
**ESR (mm/h)**								
<20	Ref.		Ref.		Ref.			
≥20	**3.55 (2.41, 5.24)**	**0**.**000**	**1.73 (1.02, 2.93)**	**0**.**042**	**1.87 (1.30, 2.69)**	**0**.**001**		
**SAA**								
<10 mg/L	Ref.				Ref.			
≥10 mg/L	**3.30 (2.08, 5.21)**	**0**.**000**			**1.78 (1.23, 2.58)**	**0**.**002**		
**WBC (×10^9^/L)**								
<9.5	Ref.							
≥9.5	**1.86 (1.06, 3.28)**	**0**.**031**			0.90 (0.50, 1.61)	0.722		
**PLT (×10^9^/L)**								
<350	Ref.		Ref.		Ref.			
≥350	**4.28 (2.63, 6.95)**	**0**.**000**	**2.71 (1.45, 5.06)**	**0**.**002**	**2.29 (1.42, 3.69)**	**0**.**001**	**1.90 (1.05, 3.44)**	**0**.**033**

*P < 0.05 was considered statistically significant (indicated in bold). SVD, serum vitamin D; HBI, Harvey-Bradshaw index; BMI, body mass index; TG, triglycerides; TC, total cholesterol; HDL, high-density lipoprotein cholesterol; LDL, low-density lipoprotein cholesterol; hs-CRP, high-sensitive C-reactive protein; ESR, erythrocyte sedimentation rate; SAA, serum amyloid A; WBC, white blood cell; PLT, platelet counts.*

Furthermore, we performed a multivariate logistic regression to establish the reduced model. According to the reduced model, penetrating disease (B3) (*OR* = 2.18; 95% CI: 1.08–4.37), HDL < 1.03 mmol/L (*OR* = 1.91; 95% CI: 1.14–3.19), ESR ≥ 20 mm/h (*OR* = 1.73; 95% CI: 1.02–2.93), and PLT ≥ 350 × 10^9^/L (*OR* = 2.71; 95% CI: 1.45–5.06) were regarded as predictors for severe vitamin D deficiency. HDL < 1.03 mmol/L (*OR* = 1.76; 95% CI: 1.14–2.72) and PLT ≥ 350 × 10^9^/L (*OR* = 1.90; 95% CI: 1.05–3.44) were also regarded as a risk factors for mild vitamin D deficiency.

## Discussion

In this study, 862 Chinese patients with CD and 576 healthy controls were selected for vitamin D deficiency assessment. 25(OH)D was determined by the gold standard assay (LC–MS/MS). Our data showed that the incidence of severe vitamin D deficiency was significantly higher in patients with CD (28.9%) than in healthy controls (9.5%). Compared with the SVD > 20 ng/mL or 10–20 ng/mL groups, inflammation, hypohemoglobin, and dyslipidemia in the SVD < 10 ng/mL group were more serious. Meantime, using multinomial logistic regression, penetrating disease, low level of HDL, and high levels of ESR and PLT were defined as predictors of severe vitamin D deficiency, while only PLT and HDL were considered as predictors of mild vitamin D deficiency.

Vitamin D status in different populations has been well-demonstrated (30). However, there is no absolute consensus on the levels to define vitamin D deficiency and sufficiency. Previous studies mainly focused on SVD < 20 ng/mL. A meta-analysis of 14 observational studies, including 938 patients with IBD demonstrated that the prevalence rates of vitamin D deficiency in CD and ulcerative colitis (UC) were only 38.1 and 31.6%, respectively (35). In our study, the prevalence rate of vitamin D deficiency in patients with CD was ∼72.4%, which was higher than that in the healthy controls (59.9%). Compared with healthy controls, the incidence of severe vitamin D deficiency in patients with CD increased by 19.4%, but this was not found for mild vitamin D deficiency. This finding suggests that separated analysis of SVD < 10 ng/mL could be valuable for Chinese patients with CD.

Compared with healthy subjects, the cholesterol level of IBD patients was lower, and this finding was more profound for patients with CD than for UC patients (36, 37). Our data suggested that vitamin D deficiency has a negative influence on cholesterol in patients with CD. Compared with the SVD > 20 ng/mL group, the cholesterol levels in the SVD < 10 ng/mL group were ∼10% lower. Although the exact mechanism remains unclear, we assumed that increased inflammatory responses induced by vitamin D deficiency could affect lipoprotein metabolism and be responsible for lipid derangement in patients with CD. Compared with the SVD > 20 ng/mL group, SAA increased by up to ∼4 times, and ESR and CRP increased ∼twofolds in the SVD < 10 ng/mL group, and these indices are now commonly used markers for predicting CD disease activity and inflammatory degrees (38, 39). The increase in some systemic inflammatory biomarkers obtained from the complete blood count (CBC), such as WBC, NE, MO, NLR, MLR, PLR, SII, and PLT, suggested changes in platelets, neutrophils, lymphocytes, and monocytes derived from peripheral blood, which have been proposed to predict the increased systemic and mucosal inflammation. Among them, PLT and its derived parameters (such as PLR or SII) change most significantly when vitamin D is deficient. There is evidence that the correlation between coagulation and inflammation can be considered as a possible pathogenesis instigator of IBD (40). PLT is well-known for its hemostasis and inflammatory amplification roles in chronic inflammation (40). Moreover, a previous study showed that lower vitamin D levels are associated with a higher platelet number (41). The reason may be that vitamin D receptors modulate megakaryocytopoiesis and platelet activation, which are calcium-dependent events (42). Thus, we assumed that vitamin D deficiency should contribute to increased coagulation and inflammation in part by increasing PLT in patients with CD. Compared with patients with normal vitamin D levels, patients with severe vitamin D deficiency had lower median hemoglobin and RBC levels. There is evidence that vitamin D may prevent anemia by supporting erythropoiesis (43).

Therefore, we believed the negative consequences are that vitamin D deficiency is responsible for the increased inflammation, PLT, and Lp(a) levels, and decreased RBC, hemoglobin, and HDL levels, and that they finally promote the progression of CD and increase the risk of some potential diseases, such as anemia, thrombosis, or cardiovascular disease in Patients with CD.

Regarding the identification of risk factors for vitamin D deficiency, our results raised a “chicken or egg” dilemma. Although some risk factors have been well-documented, it is still unclear whether they are a cause or consequence of vitamin D deficiency. Using correlation analysis, chi-square test, and univariate logistic regression analysis, HBI ≥ 8; ileocolonic (L3); penetrating disease(B3); low levels of TC, HDL-C and LDL-C; and high levels of ESR, hs-CRP, SAA, WBC, and PLT were defined as independent risk factors for severe vitamin D deficiency in patients with CD. However, vitamin D deficiency could be the combined effect of these factors. Results from multivariate logistic regression analysis finally revealed that penetrating disease, HDL < 1.03 mmol/L, ESR ≥ 20 mm/h, and PLT ≥ 350 × 10^9^/L were risk factors for vitamin D deficiency.

First, previous studies indicate a negative association between BMI and vitamin D. However, in our study, there was no significant correlation between BMI and 25(OH)D levels. Univariate logistic analysis revealed that subjects with a BMI > 24 kg/m^2^ was half less likely to develop severe vitamin D deficiency. This conflicting result may be due to the lower proportion of overweight (11.5%) or obesity (1.5%) in this study, which suggested that an appropriate fat mass helps to maintain vitamin D levels.

The most important finding of our study was that patients with hypocholesterolemia had a higher risk of vitamin D deficiency. The majority of studies suggested a direct positive association between vitamin D and HDL levels in healthy children and adolescents (16, 44). Most of the studies have found inverse correlations between TG, TC, and LDL levels and the vitamin D status of children and adolescents, while other studies mentioned significant or non-significant positive associations (16). A randomized controlled trial (RCT) on patients with polycystic ovary syndrome (PCOS) showed that the levels TGs decreased after supplementation with a low dose of vitamin D (45). A 2019 meta-analysis documented significant effects of vitamin D supplementation on TC, LDL, and TGs (19). The reason might be that vitamin D could reduce the production of TG through induction of intestinal absorption of calcium and suppression of serum parathyroid hormone (PTH) (15). High PTH levels could result in an increment in TG production. Increased calcium promotes the synthesis of bile acids from cholesterol and decreases serum cholesterol (15). Vitamin D may regulate macrophage functions in the reverse cholesterol transport of large HDL particles by taking cholesterol from macrophages (46). However, how lipid profiles affect vitamin D remains unknown yet.

Our findings showed that vitamin D was weakly positively correlated with TC, LDL, and HDL. Logistic regression analysis revealed that HDL was a risk factor for severe vitamin D deficiency. Compared with patients with normal HDL levels, CD patients with low levels of HDL were more than twice as likely to have severe vitamin D deficiency. CD patients with hypocholesterolemia displayed lower vitamin D levels and a higher incidence of severe vitamin D deficiency. The reason may be the obvious reduction in synthetic substances of vitamin D. However, there were no statistically significant differences in healthy controls. This result suggested that cholesterol can be considered a potential marker to predict vitamin D status in patients with CD but not healthy controls. Vitamin D could be more sensitive to changes in HDL than changes in LDL or TC.

The relationships between vitamin D and the severity of disease and inflammation are a hot topic that have been evaluated in some studies (21, 33, 47–49). In this study, we confirmed negative correlations between vitamin D concentration and disease activity, as reported by Jorgensen SP et al. (50), Meckel et al. (51), and Garg et al. (52). Meantime, we found that an HBI ≥ 8 may be an independent risk factor for severe vitamin D deficiency. The levels of 25(OH)D in patients with HBI ≥ 8 were significantly lower than those in other groups, and the prevalence of severe vitamin D deficiency was higher than that in other groups.

Regarding inflammatory biomarkers, vitamin D was negatively correlated with CRP, ESR, and fecal calprotectin (48, 52, 53). In our study, we confirmed that vitamin D had a negative correlation with ESR and CRP, and found that SAA and PLT may also be influencing factors of vitamin D. Logistic regression analysis revealed that patients with higher levels of ESR (*OR* = 1.73) and PLT (*OR* = 2.71) were more susceptible to severe vitamin D deficiency. Patients with higher levels of PLT were only 1.90 times as likely to develop mild vitamin D deficiency. In our study, the SVD levels and the prevalence rates of severe vitamin D deficiency in CD patients with ESR ≥ 20 mm/h or PLT ≥ 350 × 10^9^/L were significantly higher than those in the other groups, although the difference was not statistically significant in the healthy controls. Interestingly, there were also no statistically significant differences in SVD levels between CD patients with ESR < 20 mm/h, PLT < 350 × 10^9^/L, or HDL ≥ 1.03 mmol/L groups and healthy controls. These results suggested that vitamin D status might benefit from the improvement of HDL, PLT, and ESR levels. PLT might be a more significant predictor of vitamin D deficiency, and the predictive roles of ESR and PLT for vitamin D deficiency in patients with CD were not applicable for healthy controls.

Moreover, logistic regression analysis revealed that patients with penetrating disease (B3) were approximately twice as likely to develop severe vitamin D deficiency (SVD < 10 ng/mL) compared to patients with non-stricturing non-penetrating disease (B1). CD is characterized by transmural rather than superficial mucosal inflammation. Transmural inflammation often leads to fibrosis and obstructive clinical presentations, followed by micro-perforations and fistulae (54). Severe mucosal damage and reduced dietary consumption may lead to long-term malabsorption of vitamin D.

Our study still has some limitations. First, the study was conducted in Zhejiang, South China, so the participants might not represent the entire Chinese population. Second, there was a lack of vitamin D assessment based on sunlight exposure, diet, and supplementary intake. However, population mobility, a relatively large sample size, and a relatively large time-span increase the reliability of our research. Prospective cohort studies in other populations are needed to confirm our findings.

## Conclusion

The findings of this study indicate that vitamin D status in patients with CD in China is worse than expected and highlight the crosstalk among vitamin D, inflammation, and lipid metabolism. Penetrating disease, reduced HDL levels, and increased ESR and PLT levels may be considered predictors of severe vitamin D deficiency, and only HDL and PLT may be considered a predictor of mild vitamin D deficiency.

It can be concluded that patients with CD may benefit from vitamin D status monitoring. Vitamin D deficiency may contribute to increasing the risk of some potential diseases, such as anemia, thrombosis, or cardiovascular disease by reducing HDL levels and inducing inflammation. Vitamin D supplementation could be especially important in patients who exhibit penetrating disease, high levels of ESR or PLT, or low levels of HDL, and vitamin D supplementation should, in turn, improve disease behavior and systemic inflammation.

## Data Availability Statement

The raw data supporting the conclusions of this article will be made available by the authors, without undue reservation.

## Author Contributions

All authors have accepted responsibility for the entire content of this submitted manuscript and approved submission.

## Conflict of Interest

The authors declare that the research was conducted in the absence of any commercial or financial relationships that could be construed as a potential conflict of interest.

## Publisher’s Note

All claims expressed in this article are solely those of the authors and do not necessarily represent those of their affiliated organizations, or those of the publisher, the editors and the reviewers. Any product that may be evaluated in this article, or claim that may be made by its manufacturer, is not guaranteed or endorsed by the publisher.
